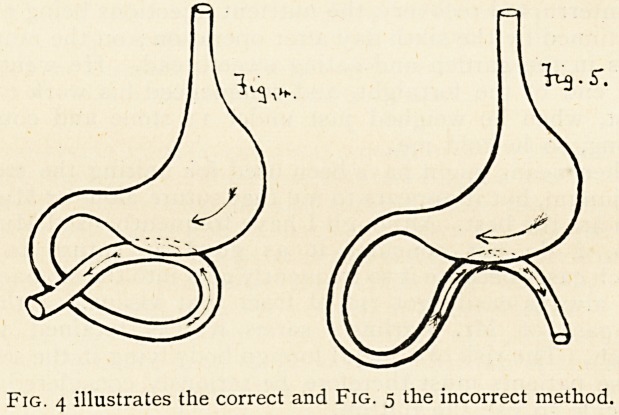# A Case of Gastro-Jejunostomy for Cancer of the Pylorus
^1^Read before the Bristol Medico-Chirurgical Society, December 11th, 1901.


**Published:** 1902-03

**Authors:** J. Paul Bush

**Affiliations:** Surgeon to the Bristol Royal Infirmary


### A CASE OF GASTRO-JEJUNOSTOMY FOR CANCER OF THE PYLORUS.[1]

BY

J. Paul Bush, C.M.G., M.R.C.S.,

*Surgeon to the Bristol Royal Infirmary.*

---

I PLACE this case of gastro-jejunostomy on record as operations of this nature are not very numerous, and the benefit derived from them is frequently of very short duration.

H. D., aged 45, came under the care of my colleague, Dr. J. E. Shaw, at the Bristol Royal Infirmary, in June, 1901, complaining of general wasting, pain in the belly, and constant vomiting; and I have to thank Dr. Shaw for giving me the earliest opportunity in his power of seeing the patient. For some two years he had suffered from flatulence and intermittent pain after food, and for a year he had noticed he was losing flesh, his normal weight being 11 stone 6 lbs. Six months before admission he was vomiting large quantities of "barmy stuff" chiefly at night, and about this time he first noticed in the vomit food he had taken days previously. This vomiting increased in frequency till admission, there had never been any considerable quantity of blood brought up.

On admission the patient weighed only 7 stone 4 lbs., having lost over 4 stone in eighteen months; he was extremely wasted and anæmic, and looked as if he had only a few days to live; he could keep down no solid food whatever, and frequently vomited liquid nourishment even when taken in small quantities; the stomach was dilated nearly down to the umbilicus, but at times a hard mass could be felt in the position of the pylorus; powerful peristalsis of the stomach could be seen, and this clinical picture Mr. Barling lays down as the clearest indication we have for surgical interference.

After consultation it was decided to do a pylorectomy. On July 2nd, the stomach having been washed out, I made a 5-inch incision in the middle line of the abdomen to 2 inches below the umbilicus (which I excised). Having explored the region with my hand in the belly cavity, I found the pylorus invaded with a tumour too large and too fixed to be removed; moreover it extended into the duodenum and a large number of infected glands could be seen and felt; I therefore had to alter my plans entirely and give up all idea of removing the whole

[1] Read before the Bristol Medico-Chirurgical Society, December 11th, 1901.

growth, and to content myself with performing a gastro-jejunostomy. I followed Von Hacker's operation as nearly as possible.

(*s*) *Stomach.* (*c*) *Transverse Colon.*
(*J*) *Jejunum.* (*a*) *Transverse Meso-colon.*
(*f*) *Mesentery.* (*b*) *Great Omentum.*

FIG. 1 and 2 represent the posterior operation (Von Hacker's).
FIG. 3 the anterior operation (Wölfler's).

The transverse colon (*c*) and great omentum (*b*) and stomach (*s*) were pushed upwards; I then tore with my finger a hole through the transverse meso-colon (*a*), and sutured these torn edges to the posterior surface of the stomach low down and as near as I could get with safety to the pylorus and the greater curvature.

In dividing the transverse meso-colon one must be very careful to select a spot without vessels, as these vessels if torn give a great deal of trouble; the stitching of the mesenteric opening to the stomach is to prevent constriction of the small gut by this hole contracting afterwards. The next step was to find the *upper* end of the jejunum (*J*), and this is best done by running the finger down the bodies of the vertebræ till the pancreas is met and one feels the reflexion of the peritoneum (the inferior duodenal fold) on to the small intestine, which is quite distinct. It is very important to make certain the highest portion of the jejunum has been found, as in one case in which I have seen this operation performed the *ileum* was by mistake joined to the stomach with fatal results from malnutrition.

The jejunum (*J*) having been milked empty and a clamp applied, it was brought up to the stomach (*s*) through the rent in the meso-colon (*a*); it was fixed in this position by posterior Lembert sutures of silk, not penetrating to the mucous coat.

A vertical incision, 2 inches long, was then made in the posterior wall of the stomach, and a corresponding incision made in the jejunum; these incisions were longer than seemed absolutely necessary, in order to avoid any possibility of the risk of subsequent over-contraction of the opening. The entire thicknesses of the cut edges were then united to each other by a whipping stitch, traversing both mucous and serous walls; this suture most efficiently stops the hemorrhage from the cut edges. Finally, the serous surfaces of jejunum and stomach in front of the anastomosis were brought together by continuous Lembert sutures, which were fastened off by tying them with the loose ends of the first set of stitches. The parts having been cleaned, the abdomen was closed in the usual way; no drain was used. The patient was so collapsed, we had to commence at once with brandy by the mouth; and I think it is important to feed the patient by the mouth at the earliest opportunity, supplementing this by regular rectal injections immediately after the completion of the operation. If vomiting should come on, a complete washing out of the stomach will usually check this, more especially if any blood should be left in that viscus.

The further details of this case need no comment. He made an uninterrupted recovery, the nutrient injections being entirely discontinued by the sixth day after operation; on the ninth day he was in the garden and eating sweetbread. He went home at the end of the fortnight, and commenced his work early in August, when he weighed just under 11 stone and could eat anything, so he told me.

Other means might have been used for uniting the stomach and jejunum, but it appears to me that suture alone or Murphy's button are the best. Although I have frequently used Murphy's button, I do not consider it as good as suture in these stomach cases because it so frequently gets into the stomach, and is not always easily got rid of from that viscus. I think in every case of Mr. Barling's series it was retained in the stomach. The risk of a metal foreign body lying in the stomach of these patients must therefore be seriously considered before we decide to use the button.

In choosing between the anterior operation (Wölfler's) or the posterior operation (Von Hacker's), I prefer fixing the jejunum to the *posterior* wall of the stomach: first, because there is very much less regurgitation of fluids from the small intestine into the stomach, on account of its position (Fig. 2); secondly, because you avoid the risk of strangulating the transverse colon (*c*) by bringing up; over and in front of it, the small intestine (*J*) with its mesentery (*f*). There is also in my opinion less chance of kinking of the jejunum by dragging it up into this forced position. Even if you avoid this, in the anterior operation tympanites is often very trying, and is usually due to the vertical pressure across the front of the colon. Thirdly, the stomach drains so very much better, more especially during the all-important period of the first days following operation, when the patient must of necessity be in bed.

I think that often the regurgitation of food mixed with bile and pancreatic juice, which in several cases has proved so troublesome, is due to the fact that the communication of the stomach with the small intestine is not well placed, *i.e.* not at the most dependent portion of the stomach. It is therefore wise to try and make this opening, on its posterior surface, towards the pyloric end, and as near the greater curvature as possible. Another cause of regurgitation is the fixing of the intestine to the stomach in the wrong direction. This ought not now to occur, as Rockwitz has pointed out that it is very important that the small intestine should be applied to the stomach in such a manner that when the opening is established between the two, the stomach contents will be passed into the intestine in the normal direction of the peristalsis of both the stomach and the jejunum; and to obtain this a half turn upwards must be made of the intestine before fixing it to the stomach, so that the proximal end of the loop is towards the cardiac end of that viscus.

FIG. 4 illustrates the correct and FIG. 5 the incorrect method.

In deciding between a gastro-jejunostomy only, or a complete removal of the pylorus and growth, we must bear in mind the importance of performing the complete operation wherever possible, because we get rid of, at any rate for a time, the absorption of septic matter from an ulcerating surface secreting foul discharge into the intestinal tract; and also because we remove a surface very prone to hemorrhage. There is also always the chance of a permanent cure.

The patient remained well and at work for four months after the operation, and was up to that time maintaining his weight well; but I have since heard that secondary deposits are developing rapidly, and that he is losing ground.[1] I trust the physicians will excuse me if I appeal to them to submit this

[1] Since the above paper was written, Mr. G. Boyd, of Portishead, has kindly furnished me with the following facts. The patient died on December 14th, of secondary deposits. The newly made channel between the stomach and jejunum continued to do its work to the last, the patient being able to eat chops and steaks and any solids he fancied up to within a few days of his death. He was thus saved the painful death by starvation, and for nearly six months after the operation he was enabled to live a useful life.

class of patient as early as possible to surgical interference, if the surgeon is to interfere, and not wait until it is only "a last chance." I feel sure the heavy mortality of this operation has been largely caused by cases being operated upon which were too advanced. Just one word of caution: I would not advise any man to undertake an operation of this nature on the living until he has frequently performed it on the dead body, as with practice the time taken can be reduced by more than one half.

## Figures and Tables

**Fig. 1 and 2 Fig. 3 f1:**
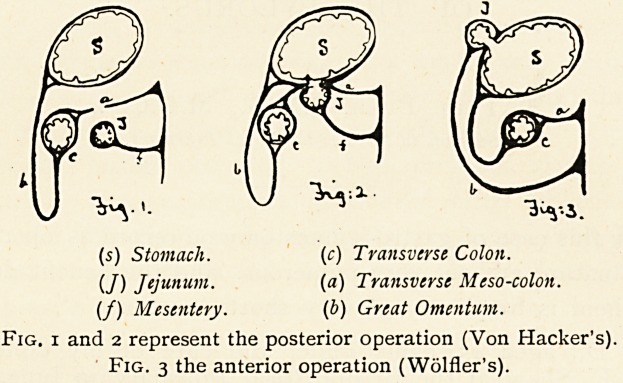


**Fig. 4 and Fig. 5 f2:**